# Therapeutic targeting of white adipose tissue metabolic dysfunction in obesity: mechanisms and opportunities

**DOI:** 10.1002/mco2.560

**Published:** 2024-05-24

**Authors:** Zi‐Han Yang, Fang‐Zhou Chen, Yi‐Xiang Zhang, Min‐Yi Ou, Poh‐Ching Tan, Xue‐Wen Xu, Qing‐Feng Li, Shuang‐Bai Zhou

**Affiliations:** ^1^ Department of Plastic and Burn Surgery West China Hospital of Sichuan University Chengdu China; ^2^ Department of Plastic & Reconstructive Surgery Shanghai Ninth People's Hospital Shanghai Jiao Tong University School of Medicine Shanghai China

**Keywords:** adipose tissue remodeling, immunometabolic dysfunction, obesity, signaling pathways, therapeutic potentials

## Abstract

White adipose tissue is not only a highly heterogeneous organ containing various cells, such as adipocytes, adipose stem and progenitor cells, and immune cells, but also an endocrine organ that is highly important for regulating metabolic and immune homeostasis. In individuals with obesity, dynamic cellular changes in adipose tissue result in phenotypic switching and adipose tissue dysfunction, including pathological expansion, WAT fibrosis, immune cell infiltration, endoplasmic reticulum stress, and ectopic lipid accumulation, ultimately leading to chronic low‐grade inflammation and insulin resistance. Recently, many distinct subpopulations of adipose tissue have been identified, providing new insights into the potential mechanisms of adipose dysfunction in individuals with obesity. Therefore, targeting white adipose tissue as a therapeutic agent for treating obesity and obesity‐related metabolic diseases is of great scientific interest. Here, we provide an overview of white adipose tissue remodeling in individuals with obesity including cellular changes and discuss the underlying regulatory mechanisms of white adipose tissue metabolic dysfunction. Currently, various studies have uncovered promising targets and strategies for obesity treatment. We also outline the potential therapeutic signaling pathways of targeting adipose tissue and summarize existing therapeutic strategies for antiobesity treatment including pharmacological approaches, lifestyle interventions, and novel therapies.

## Graphical abstract

White adipose tissue undergoes dynamic changes in obesity, including adipose tissue pathological expansion, hypoxia, adipose tissue fibrosis, immune cells infiltration and finally leads to chronic low‐grade inflammation and insulin resistance.

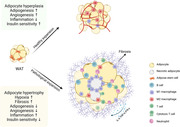

## 1 INTRODUCTION

The epidemic of obesity is now regarded as one of the most important public health problems. According to the World Health Organization (WHO), the global adult obesity rate has more than doubled since 1990, and adolescent obesity has quadrupled.^1^ In 2022, 43% of adults worldwide were overweight (body mass index [BMI] ≥ 25 kg/m^2^) and 16% were living with obesity (BMI ≥30 kg/m^2^).^1^ China is now the country with the highest number of people with overweight or obesity in the world, which places an enormous burden not only on the health system but also on the economy and society.^2^

Obesity significantly contributes to the initiation and progression of chronic conditions including chronic inflammation, insulin resistance (IR), type 2 diabetes mellitus (T2D), metabolic syndrome (MetS), cardiovascular disease (CVD), nonalcoholic fatty liver disease (NAFLD), and cancer.^3^, ^4^, ^5^ These conditions adversely affect an individual's work efficiency, quality of life, and longevity. In addition, obesity and impaired metabolic health are strongly correlated with COVID‐19‐associated complications and mortality.^6^ In this sense, obesity should be considered a global health priority that requires system‐based management and treatment. However, preventing and treating obesity are often difficult because of the multifactorial nature of this disease. Obesity has a large variety of drivers and determinants, including genetics, epigenetics, physiology, sociocultural factors, and environmental factors, which cause an energy imbalance between calories ingested and calories expended.^7^, ^8^ Therefore, further research from multiple directions is needed to reveal and understand the potential mechanisms of obesity.

Adipose tissue (AT), serving as the main energy storage site in the body, plays a crucial part in regulating overall energy balance, metabolic stability, and insulin sensitivity. The primary types of ATs are white adipose tissue (WAT) and brown adipose tissue (BAT). WAT, the main subject of this discussion, serves as the primary location for long‐term energy storage.^9^ It is identified by the existence of sizable unilocular lipid droplets that accumulate surplus metabolic energy as triacylglycerides during anabolic states and release energy to peripheral tissues in the form of free fatty acids (FFAs) under catabolic conditions.^9^, ^10^ However, in recent years, WAT has been recognized as an endocrine organ that has both metabolic and endocrine functions by regulating glucose and lipid homeostasis as well as secreting hormones (such as leptin and adiponectin) and cytokines (such as interleukin [IL]‐6, IL‐1β, and tumor necrosis factor [TNF]‐α)) to regulate many aspects of whole‐body physiology.^11^, ^12^, ^13^, ^14^, ^15^ WAT can be further divided into subcutaneous adipose tissue (SAT) and visceral adipose tissue (VAT) according to their different distributions. The development of obesity is associated with a series of changes in the structure and function of AT, referred to as “adipose tissue remodeling.”^16^ AT demonstrates notable plasticity, being able to alter its size, cellular makeup, and function in response to both physiological and pathological circumstances. This adaptation allows it to respond to environmental changes and different stress conditions effectively. However, the expandability of WAT is limited—one of the major hypotheses linking obesity to metabolic diseases. According to the AT expandability hypothesis, AT has a finite capacity for expansion. With prolonged periods of overnutrition, there comes a point where the AT reaches its storage limit for lipids. At this stage, excess lipids start being deposited ectopically, leading to metabolic irregularities and impairments in insulin sensitivity.^17^ Although both VAT and SAT mass increase during obesity, excess visceral fat is more related to metabolic deterioration and is a strong determinant of the risk for metabolic disorders, such as IR, T2D, and CVD.^18^ On the other hand, some clinical studies have suggested that SAT expansion may have a protective effect on metabolic status in individuals with obesity.^19^, ^20^ These findings suggest that various AT depots play distinct roles in the development of metabolic issues associated with obesity. Chronic overnutrition accelerates the pathological process of WAT remodeling, causing a phenotypic shift in WAT, which is characterized by dysfunctional fat cells, chronic low‐grade inflammation, and infiltration of immune cells in the stromal vascular portion.^21^, ^22^, ^23^ Dysfunction of WAT is associated with metabolic inflexibility, which ultimately leads to systemic IR and T2D.^24^, ^25^ Metabolic flexibility is the term coined by Kelley et al.^26^ as “the capacity to switch from lipid oxidation and fatty acid uptake during fasting conditions to the suppression of lipid oxidation and increased glucose uptake, oxidation, and storage under insulin‐stimulated conditions.” Nevertheless, obese individuals with IR face compromised metabolic adaptability, characterized by the incapacity to transition effectively from fat to carbohydrate oxidation upon food intake or insulin stimulus.^27^, ^28^ WAT is the predominant source of FFAs, and the capacity of AT to store and release fatty acids during feeding to fasting is an important determinant of metabolic flexibility.^25^, ^29^ Moreover, WAT dysfunction is also a characteristic of WAT senescence and is caused by age‐related metabolic inflammation.^30^ Research from significant randomized controlled trials has demonstrated that obesity hastens the aging process and contributes to age‐related cardiac and metabolic issues.^31^, ^32^

Therefore, WAT has become a major target for obesity intervention, and obtaining a complete picture of the modulation of AT during obesity is highly important. Currently, numerous studies have uncovered novel targets and strategies for treating obesity. For example, Glucagon‐like peptide‐1 (GLP‐1) receptor agonists have shown great success in obese people.^33^ In a Phase II study involving retatrutide, a triple agonist of glucose‐dependent insulinotropic polypeptide (GIP), GLP1, and glucagon, patients with obesity experienced a 25% reduction in weight.^34^ In this review, we summarize the changes in WAT in obesity and obesity‐related metabolic dysfunction at multiple levels, including cellular changes and underlying regulatory mechanisms. We also overviewed the recent therapeutic potentials such as signaling pathways, pharmacological approaches, lifestyle interventions, and novel therapies.

## 2 CELLULAR CHANGES IN OBESE WAT

WAT is a dynamic and heterogeneous organ that contains not only adipocytes but also stem cells, preadipocytes (PreAs), endothelial cells, and various immune cells.^35^ The equilibrium between these various cell types within AT plays a crucial role in preserving both energy and immune homeostasis. This balance ensures the efficient functioning of metabolic processes and the immune system, further highlighting the importance of AT in overall health. The development of obesity results from dynamic changes and crosstalk within different cell populations and eventually leads to WAT dysfunction. Here, we discuss the cellular changes and the specific role they play in WAT dysfunction in individuals with obesity.

### 2.1 Hypertrophy and hyperplasia of adipocytes

WAT is composed mainly of adipocytes, which store excess energy as triglycerides (TGs) in their cellular lipid droplets.^36^ In response to a surplus of energy, adipocytes enlarge both in size and number to accommodate the heightened requirement for lipid storage.^17^ WAT expansion occurs through two distinct mechanisms: adipocyte hyperplasia, involving an increase in cell number, and hypertrophy, entailing an increase in cell size. Hyperplasia is considered a “healthy” WAT expansion observed in “metabolically healthy” obese individuals since it is mediated by the recruitment of adipogenic precursor cells to form functional adipocytes (adipogenesis) and leads to improved outcomes with reduced inflammation and enhanced insulin sensitivity. Conversely, in adipocyte hypertrophy, cells accumulate excess TGs within lipid droplets due to caloric intake. Eventually, when TGs reach the limits of cellular and tissue expansion, adipocytes become stressed, triggering an inflammatory response as a reaction to this stress.^37^ These alterations are associated with pathological WAT expansion and dysfunction of adipocytes, leading to abnormalities in adipokines and elevated levels of circulating FFAs, resulting in a more proinflammatory adipokine profile; exacerbating obesity‐associated metabolic decline; and causing cell death, proinflammatory cytokine release, limited angiogenesis and hypoxia, which lead to WAT inflammation, dysfunction, and IR.^21^, ^38^

### 2.2 Dysregulation of adipokine secretion

AT is mainly composed of mature adipocytes, which are capable of lipid storage and, consequently, secrete additional endocrine molecules, namely, adipokines, which regulate metabolic homeostasis. In individuals with obesity, adipocytes undergo morphological and functional alterations. On the one hand, Adipocyte hypertrophy in obesity stems from various mechanisms, such as disrupted differentiation and maturation of PreAs, expansion of lipid droplets, and irregular adipocyte osmolarity sensors.^39^ On the other hand, dysregulation of proinflammatory adipokines released by hypertrophic adipocytes exacerbates AT inflammation and dysfunction. Inflamed adipocytes exhibit heightened expression levels of inflammatory cytokines like, monocyte chemoattractant protein 1 (MCP‐1), IL‐6, and TNF‐α.^37^, ^40^, ^41^, ^42^, ^43^ In turn, inflammatory cytokines may increase adipocyte size. In SAT, TNF‐α has been reported to hinder the differentiation of PreAs by inhibiting the commitment of mesenchymal stem cells to adipogenic differentiation.^44^ For instance, leptin, the first adipokine discovered through its role in regulating food intake, is oversecreted by adipocytes in individuals with obesity, and leptin resistance develops in the cells it targets.^45^ Leptin, serving as a proinflammatory cytokine, not only enhances the release of various inflammatory cytokines like TNF‐α, IL‐6, and IL‐12, but also stimulates immune cell activation and advances low‐grade inflammation.^46^

Emerging technologies, such as single‐cell (sc) and single‐nucleotide (sn) RNA sequencing (RNA‐Seq), are advancing swiftly, offering robust tools to unravel cellular diversity and showing potential in comprehending the growth and adaptability of AT in both regular and abnormal states.^47^, ^48^ Bäckdahl et al.^49^ reported that human WAT is composed of three subpopulations of mature adipocytes, only one of which is Adipo^PLIN^, which is highly sensitive to insulin according to spatial mapping of human subcutaneous WAT. Emont et al.^50^ performed scRNAseq and snRNAseq on human WAT across a range of body weights. Moreover, the white adipocyte clusters hAd4 and hAd7 tend to negatively correlate with BMI, while the hAd5 proportion is positively correlated with BMI,^50^ indicating that particular cell types are associated with a heightened susceptibility to metabolic disorders.

### 2.3 Impaired adipogenesis and differentiation

Adipose stem and progenitor cells (ASPCs), which are the cells that can differentiate into mature adipocytes, are a heterogeneous cell population that is highly important for AT homeostasis and for coordinating AT expansion and remodeling.^51^ The advent of scRNA‐seq has offered unparalleled chances to delineate the characteristics of ASPCs in both mouse and human ATs. A consensus is beginning to take shape, indicating that the ASPC fraction includes at least three subgroups: adipose stem cells (ASCs), PreAs, and adipogenesis regulators (Aregs).^52^

#### 2.3.1 Adipose stem cells

Lin^–^/SCA1^+^/CD55^+^ and Lin^–^/CD142^–^/DPP4^+^ cells are defined here as the cells that lie at the very root of adipogenic commitment and have similar molecular and functional properties.^53^, ^54^ The molecular pathways responsible for preserving the stem cell characteristics of ASCs are still being investigated. Research on murine and human ATs has revealed that transforming growth factor β (TGFβ), known as an antiadipogenic factor, can enhance the proliferative abilities of ASCs, impede adipocyte generation, and elevate the expression of ASC‐specific markers; however, blocking TGFβ signaling yields contrasting outcomes.^54^, ^55^ The antiadipogenic Wnt signaling pathway seems to be involved in the regulation of neoadipogenesis.^54^ Furthermore, the PDGF signaling pathway, as a negative regulator of adipogenesis, plays a key role in adipose commitment and maintenance of the ASC pool.^56^, ^57^, ^58^ Multiple scRNA sequencing studies conducted on VAT from healthy and obese mice have demonstrated that obesity alters the distribution of ASPC subpopulations. This may lead to the enrichment of specific ASPC subgroups with increased extracellular matrix (ECM) and immunomodulatory capabilities, alongside modified differentiation properties.^51^, ^56^, ^59^, ^60^, ^61^ It has been reported that PDGFRβ^+^ mural PreAs contribute to adipocyte hyperplasia in response to a high‐fat diet (HFD).^56^ By combining scRNA‐seq and FACS, Hepler et al.^61^ reported that the ratio of DPP4^+^ stem cells decreased during the course of HFD feeding, whereas a distinct subpopulation, PDGFRβ^+^/LY6C^+^ cells, was named fibroinflammatory progenitors (FIPs) because of their profibrogenic/proinflammatory phenotypes, which appeared to increase. This information suggests that the frequencies of ASCs and FIPs are controlled differently in vivo, particularly in relation to HFD consumption. This regulatory difference may be one of the contributing factors to the development of adipose inflammation induced by obesity.^61^

#### 2.3.2 Preadipocytes

PreAs are the precursor cells of adipocytes and exhibit a greater adipogenic capacity with lower proliferation than ASCs.^54^ The differentiation of PreAs into adipocytes is governed by an intricate transcriptional network involving regulatory factors. Key players in this process include the nuclear receptor PPARγ and different members of the C/EBP family of transcription factors.^62^ Research utilizing scRNA sequencing identifies Lin^–^/CD142^–^/ICAM1^+^ (intercellular adhesion molecule 1) cells and Lin^–^/SCA1^+^/VAP1^+^ (vascular adhesion protein 1) cells, which are designated as PreAs in a dedicated adipogenic phase.^54^ The adipogenic capacity of PreAs differs from that of depots where subcutaneous ICAM1^+^ cells have a greater adipogenic capacity than do those from visceral or omental depots.^54^, ^63^, ^64^

Research has suggested that the proportion of committed PreAs in SAT is significantly reduced in obese women.^65^ Sárvári et al.^66^ demonstrated that obesity induced by a HFD results in a notable rise in the proportion of PreAs, while the lipogenic adipocyte subgroup diminishes in mouse epididymal WAT. This suggests that HFD‐induced obesity triggers the release of various factors associated with adipogenesis, ultimately boosting the commitment of progenitors to the adipocyte lineage.^66^ Furthermore, dysregulation of the adipogenic potential of PreAs may contribute to AT dysfunction in individuals with obesity and T2D. Studies have indicated that PreAs from individuals with obesity and T2D display compromised insulin signaling. Additionally, there is a shift in their transcriptomic profile towards modified adipocyte function, characterized by a reduction in the lipogenic adipocyte subgroup and an increase in the stressed lipid‐scavenging subpopulation.^67^

#### 2.3.3 Adipogenesis regulators

Aregs were initially discovered in the SAT of mature mice, characterized by their expression of F3 (coding for CD142).^53^ Functionally, these cells showed resistance to adipogenesis in vitro and inhibited adipocyte development both in vivo and in vitro through paracrine mechanisms.^53^ Moreover, they observed that obese ob/ob mice exhibit a notably higher abundance of Aregs compared with lean mice in both subcutaneous and visceral adipose depots.^53^ In contrast, a second study revealed CD142^+^ cells in murine models to be fully adipogenic,^54^ given their distinctive transcriptomic clustering pattern. Notably, a subset of previously identified Aregs with specific expression of *Cd142, Clec11a*, and *Fmo2* was defined.^68^ Specifically, researchers have pinpointed Rspo2 as a functional controller of the P3 population, which suppresses the maturation of early progenitors via the Lgr4 receptor in HFD‐induced obesity. Consequently, elevated levels of circulating RSPO2 in mice result in AT hypertrophy and IR. However, researchers have failed to correlate the mouse P3 cluster with human subpopulations. Therefore, additional studies are needed to delineate the functions of human SAT subpopulations.

### 2.4 Infiltration and activation of immune cells

The accumulation of proinflammatory immune cells in the AT of obese individuals with T2D and impaired immune function is a significant contributor to the onset of systemic chronic, low‐grade inflammation, and metabolic complications.^22^ Thus, it is highly important to discuss the immune cell landscape of WAT in individuals with obesity to obtain a better understanding of AT inflammation.

#### 2.4.1 Macrophages

Macrophages are the first cells discovered in AT from obese mice that form CLSs surrounding dead adipocytes.^69^ In the process of obesity, adipose tissue macrophages (ATMs) not only increase in number but also exhibit phenotypic and functional switching. First, the population of ATMs increases up to 40−50% of the SVF in VAT from obese mice.^69^, ^70^ In humans, elevated macrophage infiltration, especially predominantly intraabdominal infiltration, has also been reported in obese subjects.^71^ An increase in ATM expression results in the promotion of proinflammatory cytokine secretion, monocyte recruitment and “inflammatory cross talk” with other immune cells via the chemokine receptor pathways CCR2/CCL2, CCR1/CCL5, IL‐6, interferon‐γ (IFN‐γ), TNF‐α, and so on.^72^, ^73^, ^74^, ^75^ Traditionally, macrophages are categorized as proinflammatory “classically activated” M1‐like macrophages or anti‐inflammatory “alternatively activated” M2‐like macrophages.^76^ Correspondingly, the phenotype of ATMs changes from an M2‐like state to an M1‐like state in individuals with obesity. The M1‐like phenotype is triggered by Th1 mediators like IFNγ and LPS, displaying characteristics such as TNF‐α secretion, along with the expression of iNOS and CD11c. In contrast, M2‐like macrophages, influenced by Th2 mediators like IL‐4, are recognized by their secretion of IL‐10, and the expression of arginase 1 and PPARγ in both mice and humans.^77^, ^78^, ^79^ Notably, the inflammatory phenotype switch of ATM in individuals with obesity plays an indispensable role in AT inflammation and IR.^80^ M1‐like macrophages block insulin action in adipocytes via TNF‐α, thereby inhibiting insulin signaling.^81^ Moreover, inflammatory ATMs also contribute to ECM remodeling^21^ and the recruitment and activation of other immune cells in ATs.^80^

However, categorizing macrophages into M1‐like and M2‐like subsets is simple and cannot reflect the heterogeneity of macrophages.^82^ Recent studies have unveiled that the activation of ATMs is more intricate than previously thought based on the M1/M2 paradigm. It is now recognized that in obese ATs, there are multiple populations of ATMs with distinct markers, diverse tissue distributions, unique transcriptional profiles, and functions.^83^ Kratz et al.^84^ proposed that ATMs in obese humans/mice produce a “metabolically activated (MMe)” phenotype distinct from classical activation, which involves the expression of ABCA1, CD36, and PLIN2. The metabolic activation of ATMs is steered by separate proinflammatory and anti‐inflammatory pathways. The equilibrium between these pathways dictates the overall reaction of macrophages to metabolic irregularities, leading to either a proinflammatory or an anti‐inflammatory response.^84^ Hill et al.^85^ reported that the VAT of obese mice and humans harbors various heterogeneous subsets of ATMs: CD9 ATMs in mice are found within crown‐like structures (CLSs), exhibiting lipid accumulation and a proinflammatory profile, while Ly6c ATMs are situated outside CLSs and display characteristics associated with adipogenesis.^85^ CD9 ATMs react to tissue injury, express immunomodulatory factors such as CCL2, and facilitate the recruitment of tissue‐regulatory Ly6c ATMs. The transfer of Ly6c ATMs into lean mice triggers genetic processes characteristic of typical adipocyte function.^86^ Pirzgalska et al.^87^ identified sympathetic neuron‐associated macrophages (SAMs) as a population of cells that contributes to obesity by importing and metabolizing norepinephrine. Using index and transcriptional sc sorting, Jaitin et al.^88^ described a novel and conserved Trem2^+^ lipid‐associated macrophage (LAM) subset notably arising under obese conditions, accumulating in CLSs and associated with phagocytosis, lipid catabolism, and energy metabolism. Weinstock et al.^89^ reported that obese VAT displayed expansion of a novel specialized phagocytic macrophage subpopulation that was enriched in lipid binding and metabolic processes and highly expressed the phagocytosis gene Fcgr4. A recent investigation employing sc transcriptomics and flow cytometry proposed that in obese individuals, human WAT LAMs, as previously described, play an active role in producing IL‐1β and TNF‐α. Additionally, they are believed to contribute to AT inflammation through the expression of IL‐18, CXCL8, and PDGFβ.^90^

Taken together, these data imply that the underlying mechanisms by which ATMs induce AT inflammation and IR are more complicated than previously understood.

#### 2.4.2 Innate lymphoid cells

Innate lymphoid cells (ILCs) are tissue‐resident cells that are deeply integrated into the fabric of tissues and are important for defending against a wide array of pathogens and maintaining organ homeostasis.^91^ Previous research in both mice and humans indicates that ILCs can be categorized into three distinct groups distinguished by the expression of transcription factors, cell surface markers, and effector cytokines, namely ILC1s, ILC2s, and ILC3s.^92^, ^93^, ^94^ ILC1s rely on the T‐box transcription factor Tbet for their development and produce IFN‐γ. ILC2s are reliant on GATA3 and RORα, generating IL‐5 and IL‐13. Meanwhile, ILC3s depend on the transcription factor retinoic acid receptor‐related orphan receptor γt (RORγt) and produce IL‐17 and/or IL‐22. ILC1s, ILC2s, and ILC3s reflect the functions of CD4^+^T helper (Th)1, Th2, and Th17 cells, respectively.^93^ Studies using mouse models have demonstrated that diet‐induced obesity leads to adipose‐resident ILC1 accumulation via IL‐12 production, drives M1‐like macrophage polarization through targeted cytotoxicity and induces obesity‐associated IR through IFN‐γ secretion.^95^, ^96^ Moreover, adipose ILC1s are increased in obese T2D patients and promote adipose fibrogenesis, CD11c^+^ macrophage activation and the TGF‐β1 pathway in adipocytes.^97^ On the other hand, evidence has revealed that ILC2s are abundant in the lean state and play a role in preserving metabolic balance by expressing IL‐33, whereas ILC2s are reduced in the WAT of obese mice and humans. This reduction leads to disrupted beige adipocyte formation, as well as the gathering of eosinophils and M2‐like macrophages in an IL‐4/IL‐13‐dependent manner, resulting in obesity characterized by enduring, low‐grade type 1 inflammation.^98^, ^99^, ^100^ Recent scRNA‐seq research demonstrated an elevation of ILC3s and ILC precursor (ILCP)‐like cells in the WAT of obese human subjects. It was observed that obese ILC3s potentially act as regulators of AT inflammation through TNFSF13B and MIF, affecting macrophages, dendritic cells (DCs), and monocyte subsets, suggesting that ILC3s likely have significant involvement in the biological processes of human WAT.^90^

#### 2.4.3 Neutrophils

Generally, neutrophils are considered the major leukocytes that protect against infections by releasing lytic enzymes and reactive oxygen species (ROS) and producing neutrophil extracellular traps.^101^ The number of neutrophils in the circulatory system is significantly increased in individuals with a higher BMI and an activated CD66b phenotype, and these cells stimulate the NF‐κB signaling pathway via ROS and proinflammatory cytokine production.^102^, ^103^, ^104^, ^105^, ^106^, ^107^ Moreover, neutrophils are the initial immune cells to infiltrate AT, once activated, they release inflammatory molecules that attract macrophages and other immune cell types to the site of inflammation.^108^ In the early stage of HFD feeding (after 3 days), the number of neutrophils in mouse periepididymal fat significantly increased 3.5‐fold compared with that on day 0, and these cells directly interact with adipocytes through complex formation between neutrophil CD11b/Mac1 and adipocyte ICAM‐1.^108^ Activated neutrophils further promote AT inflammation by producing IL‐1β and TNF‐α.^109^, ^110^, ^111^ On the other hand, obese ATs can also produce chemotactic factors such as IL‐8 to recruit neutrophils.^112^ Neutrophils show increased production of elastase (NE), and NE null (Ela2(^−/−^)) mice exhibit improved insulin sensitivity, inflammation, and energy expenditure.^113^, ^114^

#### 2.4.4 Dendritic cells

DCs act as antigen‐presenting cells that present antigens to naïve T cells; they are considered the bridge between the innate and adaptive immune systems and play a key role in obesity‐induced inflammation.^115^, ^116^ Under homeostatic conditions, there are two main DC lineages: antigen‐presenting classical DCs (cDCs) and plasmacytoid DCs.^117^ cDCs are the major tissue‐resident DCs capable of presenting antigens and producing cytokines and chemokines for pathogen elimination.^115^ cDCs can be further divided into two subsets: CD103^+^ cDC‐1s (cDC1s) and CD11b^+^ cDC‐2s (cDC2s). Stimulation of the Wnt/β‐catenin pathway in cDC1s triggers the generation of IL‐10, whereas the presence of PPARγ in cDC2s hinders the initiation of local inflammatory reactions.^118^, ^119^ It has been reported that DCs accumulate in the AT of obese humans and HFD‐fed mice, exacerbating the inflammatory response and causing IR.^120^, ^121^ Interestingly, recent scRNAseq analysis revealed three distinct WAT DC populations, cDC1, cDC2B, and cDC2A, whose populations accumulate in the WAT of obese humans.^90^ Although the frequency of DC did not change, the density of DC in obese subjects was greater than that in lean subjects and was correlated with an increase in BMI. The loss of DCs in several mouse models (*Flt3l* ^−^/^−^ and *Csf2* ^−^/^−^ mouse models) prevents HFD‐induced weight gain and IR.^122^, ^123^ In addition, DCs in obese ATs induce Th1 and Th17 cell activation and proliferation via IFNγ and IL‐17 production, which create a proinflammatory environment.^120^, ^124^ Furthermore, IFNγ secreted by Th1 cells can stimulate the expression of MHC‐II, establishing a feedback loop that amplifies Th1 cell responses and worsens AT inflammation.^125^, ^126^

#### 2.4.5 B cells

B cells play an indispensable role in the adaptive immune system by exerting a specific immune response and developing immunological memory through cytokine and antibody secretion.^127^ Typically, B cells are identified by markers such as CD19 and CD45R (B220) in flow cytometry. They can be classified into B1 and B2 cells, primarily distinguished by their origin, developmental pathways, anatomical locations, and dependence on T‐cell assistance for antibody synthesis.^128^ B2 cells are conventional B cells that can secrete proinflammatory IgG molecules (IgG2c) and cytokines (including MCP1, TNF, IL‐6, IL‐8, and IFNγ,) to induce immunometabolic dysfunction in AT.^128^, ^129^, ^130^ Obesity results in a significant buildup of B cells in VAT, notably increasing the ratio and total count of class‐switched mature IgM^–^IgD^–^IgG^+^ B2 cells.^131^ In the epididymal VAT of obese mice, B2 cells secrete proinflammatory cytokines like INF‐γ and IL‐6, modulating the activation of T cells and macrophages within VAT. Conversely, a lack of B cells in mice fed a HFD leads to reduced inflammatory cytokine production from epididymal VAT and lessened IR induced by the HFD.^129^, ^132^, ^133^, ^134^ Consistent with findings in mice, B2 cells present in the circulation of obese individuals and those with obese diabetes produce higher levels of proinflammatory cytokines IL‐6 and TNF‐α compared with those from healthy individuals.^135^ Furthermore, the infiltration of B cells in AT is linked to heightened IgG production, leading to elevated levels of proinflammatory IgG2c in the serum and epididymal VAT of obese mice.^129^ In humans, IR correlates with distinct IgG autoimmune antibodies, indicating that B cells play a role in IR through (self)antigen‐specific targets.^129^, ^136^, ^137^ B‐1 cells are found in ATs and represent innate‐like B cells that generate IgM and IL‐10, fostering an anti‐inflammatory reaction even in the absence of antigens. Studies have shown a decrease in the quantity of B‐1 cells during obesity. Moreover, experiments have illustrated that transferring B‐1 cells can mitigate VAT inflammation, glucose intolerance, and IR in mice fed a HFD.^138^, ^139^ Recently, a distinct IL‐10‐producing B‐cell population, regulatory B cells (“Breg” cells), which are capable of suppressing AT inflammation, was described.^140^ Breg cells were found to be diminished in ATs of both obese mice and humans, displaying decreased IL‐10 production.^140^ Moreover, recent investigations have uncovered a new subset of B cells known as T‐bet^+^ B cells in the contexts of aging and obesity. These cells, termed CD27^−^IgD^−^ double negative B cells, are referred to as age‐associated B cells.^141^, ^142^ T‐bet^+^ B cells accumulate in ATs and exacerbate metabolic disorders during obesity.^143^ Furthermore, the transmission of serum or purified IgG from mice fed a HFD reinstates metabolic disorders in T‐bet^+^ B‐cell‐deficient mice, underscoring that IgG derived from T‐bet^+^ B cells serves as a significant mediator of inflammation in obesity.^143^

#### 2.4.6 T cells

T cells are essential components of the adaptive immune system, broadly classified into various subsets: CD8^+^ T cells, IFN‐γ‐producing CD4^+^ T (Th1) cells, IL‐4‐secreting Th2 cells, IL‐17‐releasing Th17 cells, and IL‐10‐generating Foxp3^+^ T regulatory cells (Tregs).^144^ Like B cells, T cells have been found to accumulate in obese ATs and promote inflammation.^145^

Th1 cells exert proinflammatory effects by expressing the transcription factor T‐bet and producing IFN‐γ, IL‐2, and TNFα.^146^ Th1 cells are abundantly present in both SAT and VAT in HFD‐fed mice compared with control diet‐fed mice.^125^, ^147^ Using IFN‐γ^−/−^ and T‐bet^−/−^ obese mouse models to block Th1 function led to decreased AT inflammation and improved glucose tolerance.^125^, ^148^, ^149^ Notably, increased expression of leptin, PPARγ, and CAAT/enhancer‐binding protein α (C/EBPα) was found in the WAT of T‐bet KO mice.^149^

Th2 cells are identified by the presence of the transcription factor GATA3 and are known for primarily producing IL‐4, IL‐5, and IL‐13 through the activation of STAT5 and STAT6, additionally generating the anti‐inflammatory cytokine IL‐10.^150^ Research indicates that Th2 cells exert an anti‐inflammatory function in obesity, with their population decreasing in the VAT of mice fed a HFD, whereas the transferring CD4^+^ T cells into lymphocyte‐free Rag1‐null mice with DIO reverses weight gain and IR primarily through the action of Th2 cells.^151^

Th17 cells express RORγt and STAT3 to stimulate inflammatory processes through the secretion of IL‐17. Th17 cells accumulate in the AT and circulatory system in both obese mice and humans.^120^, ^152^, ^153^, ^154^ A study revealed an elevated presence of Th17 cells and heightened levels of IL‐1β, IL‐6, and IL‐17 in VAT from metabolically unhealthy obese individuals, and P2X7R agonists like ATP were shown to induce a proinflammatory environment favoring Th17 cell differentiation within VAT, resembling the milieu observed in obese patients with metabolic alterations.^155^

Tregs exert immunoregulatory effects by producing the inhibitory cytokines IL‐10 and TGFβ, which are maintained by tissue IL‐33 levels to maintain metabolic homeostasis.^156^, ^157^, ^158^ Treg numbers decrease in obese mice and humans, and adoptive transfer of CD4^+^FoxP3^+^ Tregs significantly improves insulin sensitivity and diabetic nephropathy.^159^ Furthermore, PPARγ agonists such as thiazolidinedione (TZD), 5‐aminosalicylic acid, and IL‐33 were shown to regulate VAT Treg accumulation and improve IR in obese mouse models.^157^, ^160^, ^161^, ^162^

A study has also confirmed that Treg numbers are reduced in obese human ATs and that increased IFNγ production may play an important role in AT Treg loss and obesity‐associated inflammation in humans.^163^ CD8^+^ T cells, recognized as cytotoxic T lymphocytes, proliferate in quantity during obesity and exhibit an elevated capability to release cytokines (IFNγ) and cytotoxic substances (perforin and granzymes) while engaging in direct cell‐to‐cell contact.^164^, ^165^ Studies in both mice and humans have revealed that infiltration of CD8^+^ T cells in the VAT of obese mice recruits macrophages and promotes AT inflammation, highlighting a crucial role for the crosstalk between CD8^+^ T cells and other cells in ATs.^166^, ^167^, ^168^

**FIGURE 1:**
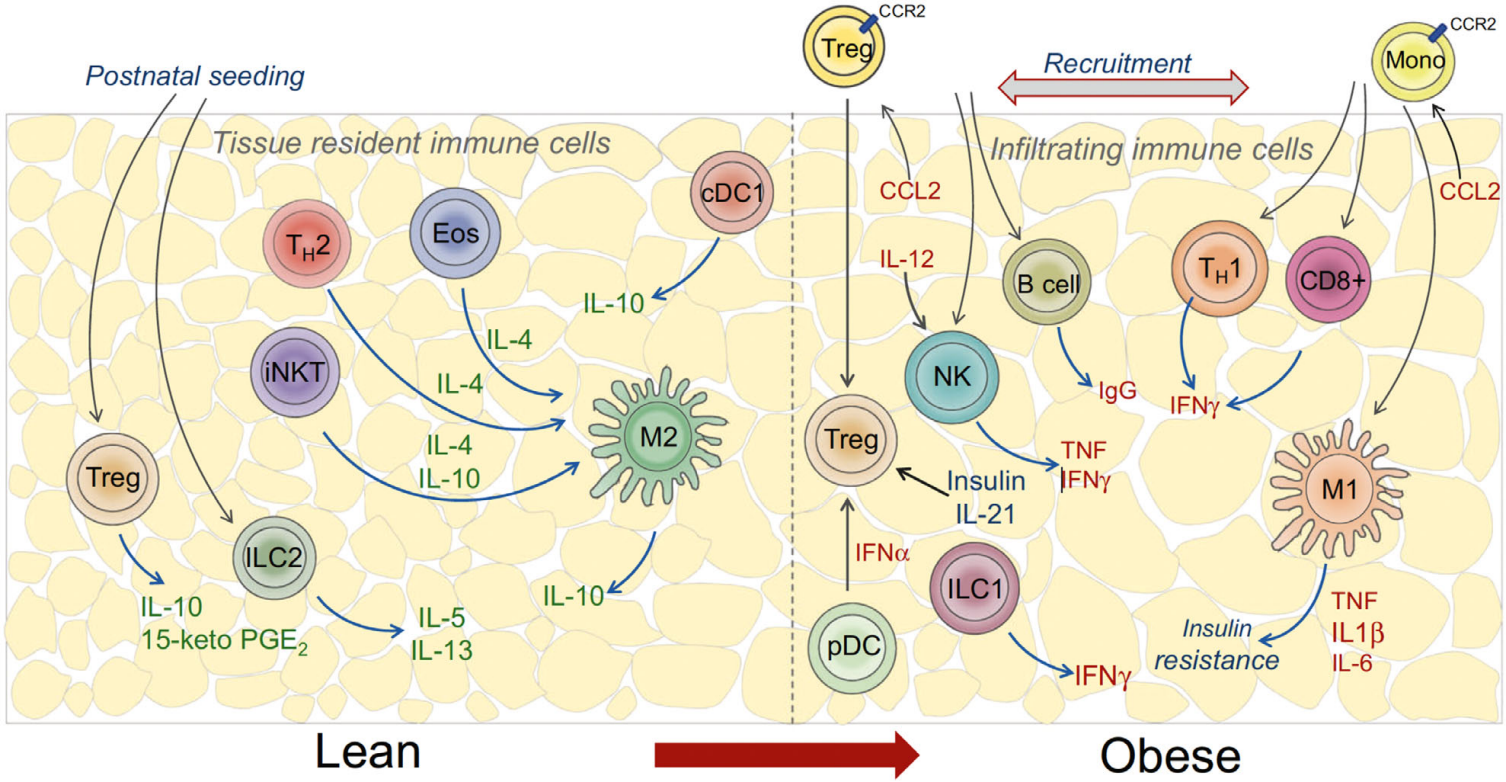
Immune spectrum in lean and obese adipose tissue. Lean adipose tissue is characterized by the presence of immune cells that promote an anti‐inflammatory environment. These immune cells, including Treg cells, eosinophils, TH2 cells, M2 macrophages, ILC2s, NKT cells, and γδT cells, play a crucial role in maintaining insulin sensitivity. Importantly, these immune cells establish residence in adipose tissue early in life. However, in obesity, the balance is disrupted as inflammatory immune cells are recruited. These include M1 macrophages, TH1 cells, CD8^+^ T cells, NK cells, ILC1s, and B cells. The displacement of anti‐inflammatory immune cells is facilitated by signals such as hypoxic and ER stress responses, as well as chemokines like MCP‐1/CCL2. This image is reproduced with permission from Man et al.^169^ http://creativecommons.org/licenses/by/4.0/

Taken together, the interactions between innate and adaptive immune cells, as well as communication with adipocytes and other cell types in AT, contribute to the complex pathogenesis of obesity‐associated IR. We summarize the cellular changes and their specific contribution to WAT dysfunction in obesity (Table 1). Here, Man et al.^169^ made a great schematic representation of the immune spectrum in lean and obese AT (Figure 1). Obesity‐induced inflammation persists chronically and leads to IR. This shift in the immune cell profile and prolonged inflammation can have detrimental effects on metabolic health. Indeed, chronic AT inflammation and IR underlie many of the comorbidities observed in obese individuals.

**TABLE 1:** Cellular changes in WAT during obesity.

Cell types	Changes in obesity	References
Mature adipocyte	Hypertrophy ↑ Release proinflammatory adipokines and cytokines ↑ Specific cell clusters found in obesity: AdipoPLIN; hAd4, hAd5, hAd7	^43^, ^44^, ^45^, ^46^, ^47^, ^49^, ^50^
ASPCs		
ASCs	Subgroups which exhibit enhanced ECM and immunomodulatory capacities as well as altered differentiation abilities ↑ DPP4^+^ stem cells ↓ FIPs ↑	^51^, ^56^, ^59^, ^60^, ^61^
PreAs	Proportion of committed preadipocytes ↓ Adipogenic potential ↓	^65^, ^66^, ^67^
Aregs	Fraction of Aregs (CD142^+^ABCG1^+^ ASPCs) ↑ Adipose tissue hypertrophy and insulin resistance ↑	^53^, ^54^, ^68^
Immune cells		
Macrophages	ATM number ↑ Phenotype switch from M2 to M1 Proinflammatory cytokines ↑ Monocyte recruitment ↑ Inflammatory cross‐talk ↑ Newly identified population in obesity: MMe‐ATMs, CD9 ATMs, SAMs, LAMs, novel specialized Phagocytic macrophage	^69^, ^70^, ^71^, ^72^, ^73^, ^74^, ^75^, ^76^, ^77^, ^78^, ^79^, ^80^, ^81^, ^82^, ^83^, ^84^, ^86^, ^87^, ^88^, ^89^, ^90^
ILCs	Adipose‐resident ILC1s (proinflammation) ↑ ILC2s (anti‐inflammation) ↓ ILC3s and ILCP‐like cells ↑	^90^, ^95^, ^96^, ^97^, ^98^, ^99^, ^100^
Neutrophils	Proportion↑ Proinflammatory signal ↑	^102^, ^103^, ^104^, ^105^, ^106^, ^107^, ^108^, ^109^, ^110^, ^111^, ^112^, ^113^, ^114^
DCs	Density ↑ Inflammation ↑ Insulin resistance ↑ Activation of Th1 and Th17 cells to induce inflammation	^116^, ^117^, ^118^, ^119^, ^120^, ^121^, ^122^, ^123^, ^124^, ^125^, ^126^
B cells	B1 cells (anti‐inflammation) ↓ B2 cells (proinflammation) ↑ Proinflammatory cytokines ↑ Bregs ↓ T‐bet^+^ B cells ↑	^127^, ^128^, ^129^, ^130^, ^131^, ^140^, ^141^, ^142^, ^143^
T cells	Th1 cells (proinflammation) ↑ Th2 cells (anti‐inflammation) ↓ Th17 cells (proinflammation) ↑ Tregs ↓ CD8^+^ T cells ↑	^143^, ^144^, ^145^, ^146^, ^147^, ^148^, ^149^, ^150^, ^151^, ^152^, ^153^, ^154^, ^155^, ^156^, ^157^, ^158^, ^159^, ^160^, ^161^, ^162^, ^163^, ^164^, ^165^, ^166^, ^167^, ^168^

↑indicates increased, ↓indicates decreased.

Abbreviations: Areg, adipogenesis regulator; ASC, adipose stem cells; ASPC, Adipose stem and progenitor cell; ATM, adipose tissue macrophage; DC, dendritic cell; ECM, extracellular matrix; FIP, fibroinflammatory progenitor; ILC, innate lymphoid cell; ILCP, ILC precursor; LAM, lipid‐associated macrophage; MMe, metabolically activated; PreA, preadipocyte; SAM, sympathetic neuron‐associated macrophage; Treg, T regulatory cell.

## 3 MECHANISMS UNDERLYING METABOLIC DYSFUNCTION OF WAT IN OBESITY

**FIGURE 2:**
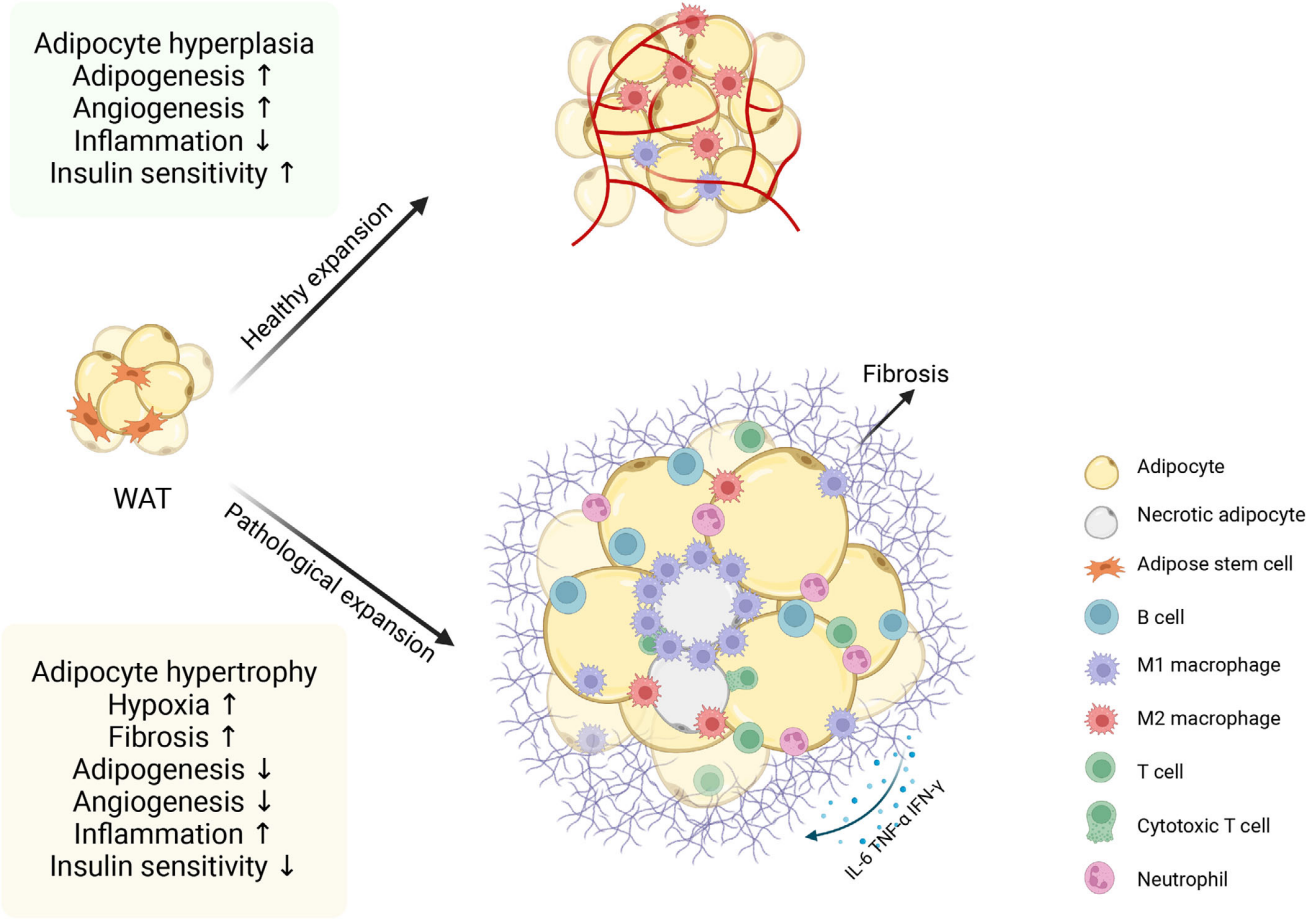
Distinct modes of WAT expansion associate with metabolic health. Different expansion mechanisms of white adipose tissue upon overnutrition lead to distinct metabolic outcomes. Healthy expansion through adipocyte hyperplasia is associated with improved adipogenesis and angiogenesis, decreased inflammation, and improved insulin sensitivity. Pathological expansion through adipocyte hypertrophy is related to limited angiogenesis and promotes WAT hypoxia, fibrosis, and inflammation, ultimately leading to obesity‐associated metabolic complications.

The changes of different cell subpopulation during obesity eventually lead to a series of changes in the structure and function of WAT, referred to as “adipose tissue remodeling,”^16^ including WAT fibrosis, inflammation, endoplasmic reticulum (ER) stress, ectopic lipid accumulation and IR (Figure 2). Here, we discuss the underlying mechanisms of metabolic dysfunction in obese WAT.

### 3.1 Fibrosis and ECM remodeling

At the early stage of WAT expansion in the obese state, hypertrophic adipocytes create localized WAT microhypoxic areas because of exacerbated oxidative stress and impaired mitochondrial respiration,^170^ and the insufficient oxygen supply of WAT results in the activation of hypoxia‐inducible factor‐1α (HIF‐1α),^171^ which subsequently regulates hypoxia‐associated genes, such as leptin and VEGF, which both play critical roles in the initiation of angiogenesis.^172^, ^173^ In particular, WAT blood flow in obese subjects is approximately 30−40% lower than that in lean subjects,^174^ and the reduction in blood flow with pathological blood vessel formation, in turn, exacerbates hypoxia. Furthermore, hypoxia within hypertrophic adipocytes triggers collagen accumulation and fibrosis in AT depots, a condition known as excessive ECM production, which arises due to an imbalance between the creation and breakdown of ECM fibrillar elements like collagen I, III, and VI.^175^ Notably, in individuals with obesity, hypoxia has been identified as a major initiating factor for ECM production by activating HIFα and subsequently inducing an alternative transcriptional program to increase the synthesis of ECM components, ultimately leading to WAT fibrosis.^175^, ^176^

### 3.2 Inflammation and oxidative stress

The rapid expansion of WAT in obesity ultimately leads to a metabolic inflammatory response and is strongly associated with systemic IR, namely, metaflammation.^176^ However, the specific inflammatory trigger in AT remains unidentified but can be initiated by several potential intrinsic signals, including a gut‐derived substance, dietary component or metabolite, adipocyte death, hypoxia, and mechanical stress.^177^ Under conditions of overnutrition, the lipid metabolism of hypertrophic adipocytes is dysregulated, and insulin signaling pathways are impaired, leading to increased lipolysis rates and excess FFA production. FFA promotes downstream NF‐κB signaling by binding to Toll‐like receptors (TLRs), such as TLR4 and TLR2.^178^, ^179^ Once activated, NF‐κB can increase the synthesis and secretion of adipokines such as IL6 and MCP1/CCL2 (C‐C chemokine ligand 2), especially MCP‐1, which can recruit proinflammatory macrophages to AT.^180^ The infiltrated macrophages can further enhance the inflammatory response through crosstalk with adipocytes by secreting inflammatory cytokines, such as TNFα, eventually leading to a stable chronic inflammatory state.^16^ On the other hand, adipocyte death in WAT from obese mice and humans strongly stimulates macrophage infiltration and phagocytosis.^181^ In particular, macrophages have been observed to cluster and create CLSs encircling necrotic adipocytes in both obese mice and humans.^181^, ^182^, ^183^, ^184^ The NOD‐like receptor family is also activated by necrotic adipocytes, leading to activation of the NALP3 inflammasome in macrophages to induce subsequent IL‐1β and IL‐18 secretion via caspase 1.^185^ In addition, hypoxia, as discussed above, is considered an initiator of WAT inflammation in obese rodents and patients and is associated with the upregulation of inflammatory cytokines, including macrophage migration inhibitory factor (MIF), the matrix metalloproteinases MMP2 and MMP9, IL‐6, Angplt4, PAI‐1, VEGF, and leptin.^171^, ^186^, ^187^, ^188^, ^189^ Overall, the pathological expansion of WAT promotes proinflammatory immune cell infiltration and increases inflammatory cytokine secretion, which results in an inflammatory phenotype and associated metabolic disorders.

### 3.3 ER stress and the unfolded protein response

One important cellular process that has been implicated in AT dysfunction is ER stress. The ER is responsible for the proper folding and processing of newly synthesized proteins. Various factors, including nutrient overload, oxidative stress, inflammation, and alterations in lipid metabolism, can disrupt protein folding in the ER lumen, leading to the accumulation of misfolded or unfolded proteins—a condition referred to as ER stress.^190^ When cells encounter ER stress, they engage in a sophisticated signaling cascade termed the unfolded protein response (UPR). The UPR aims to restore ER homeostasis by attenuating protein synthesis, increasing ER chaperone expression, and promoting the degradation of misfolded proteins. However, if ER stress persists or becomes overwhelming, the UPR can switch from a prosurvival to a proapoptotic mode, triggering cell death.^191^ Emerging evidence suggests that obesity can act as both a cause and a consequence of an uncontrolled ER stress response.^192^, ^193^ In AT, chronic ER stress and dysregulated UPR signaling have been associated with the development of metabolic dysfunction. ER stress in adipocytes impairs insulin signaling, promotes inflammation, disrupts lipid metabolism, and leads to the release of proinflammatory cytokines and adipokines. In mice, a HFD intensified ER stress, leading to persistent inflammation within AT. Chemical chaperones such as 4‐PBA and TUDCA altered metabolic dysregulation and reduced the levels of inflammatory cytokines.^194^ Another research study demonstrated that the targeted removal of the ER chaperone GRP78 in macrophages shielded mice from AT inflammation and IR induced by a HFD.^195^ Furthermore, in obese individuals, markers of ER stress in adipocytes are significantly correlated with BMI or body fat percentage.^196^

In summary, ER stress and the UPR play critical roles in the development of metabolic dysfunction in AT. Understanding the underlying mechanisms involved in ER stress and UPR signaling may provide valuable insights for developing therapeutic interventions aimed at mitigating or preventing metabolic disorders associated with AT dysfunction.

### 3.4 Ectopic lipid accumulation and IR

When the cell and tissue expansion capacity is exhausted and further anabolic demands cannot be managed, AT becomes inefficient in storing energy.^170^ Thus, lipids can no longer be effectively extracted from the circulatory system. Consequently, surplus circulating FFAs, dietary lipids, ROS, and proinflammatory cytokines released by enlarged ATs will infiltrate non‐adipose organ cells like the liver, pancreas, and muscle, leading to ectopic fat deposition and inducing lipotoxicity.^197^, ^198^ The toxic lipids subsequently disrupt various cellular organelles like ER, mitochondria, and lysosomes, resulting in cellular dysfunction, systemic disturbances, cell death, and further interference with insulin sensitivity and glucose regulation.^199^ Dysregulated organelles produce an overabundance of ROS which instigates inflammation, leading to a widespread inflammatory response. Finally, obesity is associated with a state of chronic low‐grade inflammation because of the inflammatory mediators released by ectopic fat depots and infiltration of macrophages.^200^

**FIGURE 3:**
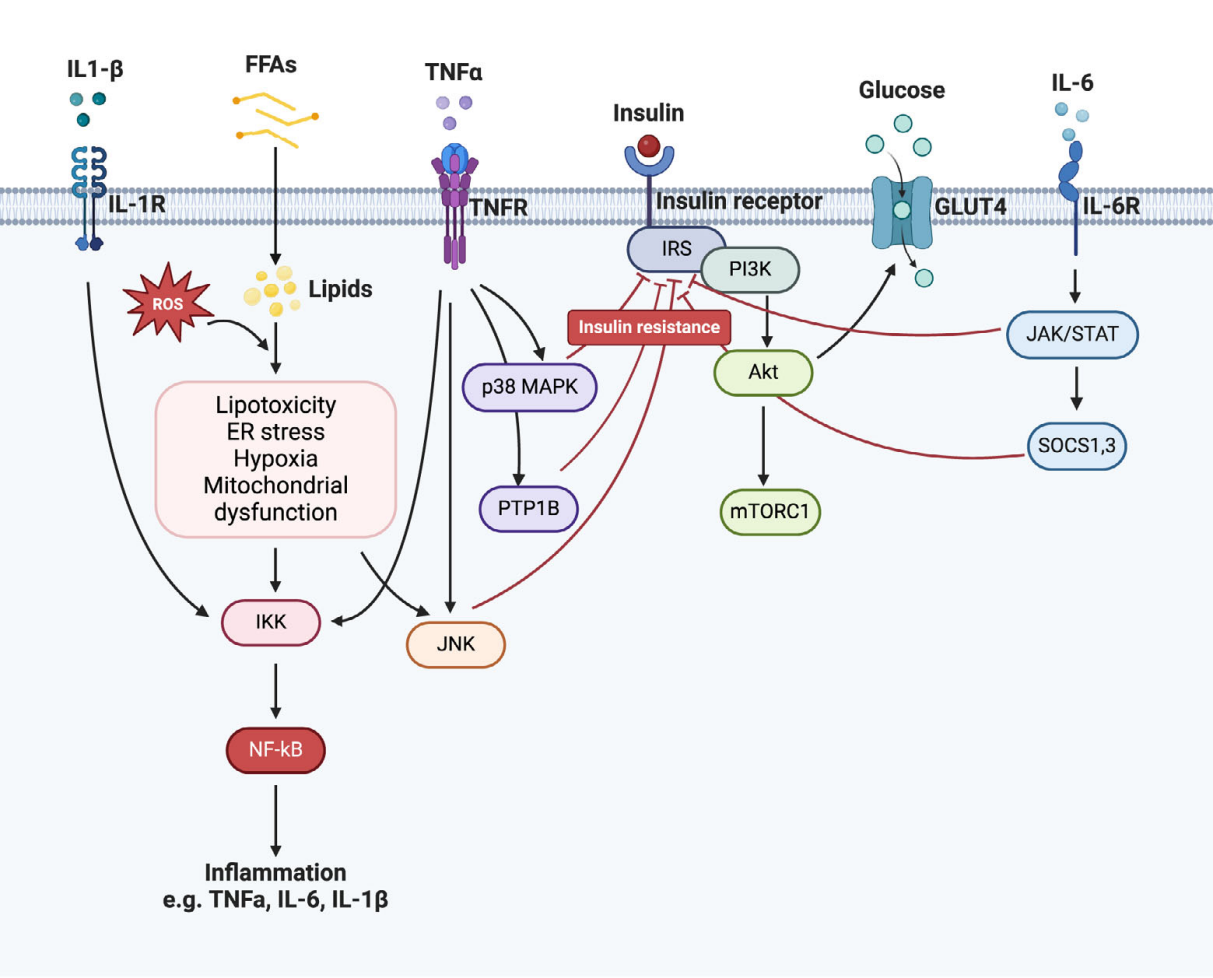
Potential mechanisms of obesity‐induced insulin resistance. Chronic overnutrition induces dysfunctional white adipose tissues to release free fatty acids (FFAs), reactive oxygen species (ROS), and proinflammatory cytokines. Thus, surplus FFAs and other lipids accumulate in the cells of peripheral organs, such as muscle and liver; cause lipotoxicity; dysregulate organelles, including mitochondria and lysosomes; and inhibit the function of insulin through various signaling pathways targeting IRS kinases, including NF‐κB, P38 MAPK, JNK, and JAK–STAT, and so on, leading to insulin resistance in adipose tissues, muscles, and the liver. Dysfunctional organelles in turn increase the release of excess FFAs, proinflammatory cytokines, and ROS. Overall, obesity promotes chronic low‐grade systemic inflammation and the development of insulin resistance, eventually leading to immunometabolic dysfunction.

The persistent, low‐grade inflammation of WAT gradually progresses to systemic inflammation and ultimately leads to IR, resulting in systemic dysregulation. IR is characterized by an inadequate response to insulin circulating in the body, particularly within insulin‐targeted tissues like AT, the liver, and skeletal muscles.^32^ Numerous mechanisms have been suggested to explain IR induced by obesity, including lipotoxicity, ER stress, mitochondrial dysfunction, oxidative stress, hypoxia, and disruption of the insulin signaling pathway, among which, obesity‐related inflammation emerges as a significant contributor to IR^201^ (Figure 3).

During long periods of excessive caloric intake, dysfunctional ATs release FFAs, ROS, and proinflammatory cytokines and subsequently activate the NF‐κB and P38 mitogen‐activated protein kinase (MAPK) signaling pathways to enhance ER stress and the secretion of proinflammatory cytokines.^202^ On the other hand, AT from obese T2D patients also secretes proinflammatory adipokines, such as MCP‐1, TNF‐α, IL‐1β, and IL‐6, which can recruit additional proinflammatory immune cells, including M1 macrophages, neutrophils, B‐2 cells, CD8^+^ T cells, and IFN‐γ^+^ CD4^+^ T cells.^14^

In obesity, the upregulation of TNF‐α stimulates the activation of inhibitor of IκB kinase (IKK)‐β and MAPKs (such as p38, c‐Jun N‐terminal kinase [JNK], and extracellular signal‐regulated kinase [ERK]). These enzymes directly target serine residues on the insulin receptor substrate (IRS) protein, hindering its tyrosine phosphorylation in an NF‐κB‐dependent manner. This cascade ultimately culminates in IR within insulin‐targeted tissues.^203^, ^204^ Moreover, TNF‐α increases the expression of protein‐tyrosine phosphatase (PTP)1B, which disrupts insulin signaling by dephosphorylating tyrosine residues on the insulin receptor and IRS‐1/2. Similarly, IL‐1β triggers the NF‐κB and MAPK pathways through the IL‐1β receptor, leading to impaired insulin signaling by phosphorylating serine residues on IRS1/2.^205^ IL‐6 stands as another significant inflammatory mediator contributing to IR by activating the Janus kinase‐signal transducer and activator of transcription (JAK–STAT) signaling pathway. This activation results in elevated expression of suppressor of cytokine signaling 1 (SOCS1) and SOCS3 proteins, which, in turn, decrease the expression of glucose transporter‐4 (GLUT4) and IRS‐1, further exacerbating IR.^206^

In individuals with obesity‐related IR, the pancreas also undergoes several changes, which can impact insulin secretion and function. Initially, when IR develops due to excess adiposity, pancreatic β‐cells compensate by increasing insulin production and secretion to overcome the decreased sensitivity of peripheral tissues to insulin.^207^ Over time, β‐cells no longer fully compensate for increasing IR, and chronic exposure to high levels of FFAs and hyperinsulinemia associated with obesity can lead to β‐cell dysfunction, which in turn rapidly raises blood glucose levels, eventually leading to the development of T2D.^208^ Furthermore, the chronic low‐grade inflammatory state in obesity results in the infiltration of immune cells, such as macrophages, into the pancreatic islets via the release of proinflammatory cytokines (e.g., TNF‐α and IL‐1β), which can impair β‐cell function and survival.^209^

Traditional beliefs hold that VAT, which has enhanced metabolic activity, is a major contributor to IR,^20^, ^210^, ^211^ where FFAs, products of lipolysis and adipokines can be directly drained to the liver through the portal vein, leading to IR. However, visceral fat accounts for only a small portion (approximately 10−20%) of the overall body fat, and the majority of FFAs are contributed by SAT, which calls into question the contribution of VAT to IR.^212^ Numerous researchers propose that subcutaneous truncal AT plays a pivotal role in the onset of IR.^213^, ^214^, ^215^, ^216^, ^217^ In contrast, a greater subcutaneous thigh fat mass may have a protective effect, which is associated with favorable glucose and lipid levels, as well as a lower incidence of diabetes and dyslipidemia.^218^, ^219^ Thus, the function of WAT in different depots of body compartments may be heterogeneous and may play different roles in IR. Shifting attention from VAT only to the functional heterogeneity of different WAT depots can help further understand the association between obesity and IR.

Overall, obese AT initiates inflammatory and insulin‐resistant conditions through the excessive secretion of FFAs, ROS, and proinflammatory cytokines; the dysregulation of several cellular organelles, which causes systemic dysfunction and enhances systemic inflammation; and the disruption of insulin sensitivity, ultimately leading to immunometabolic dysfunction and increasing the possibility of generating several chronic metabolic diseases. Understanding these dynamics is crucial for developing targeted interventions to address the metabolic consequences of obesity.

## 4 THERAPEUTIC POTENTIALS OF TARGETING METABOLIC DYSFUNCTION WAT IN OBESITY

**FIGURE 4:**
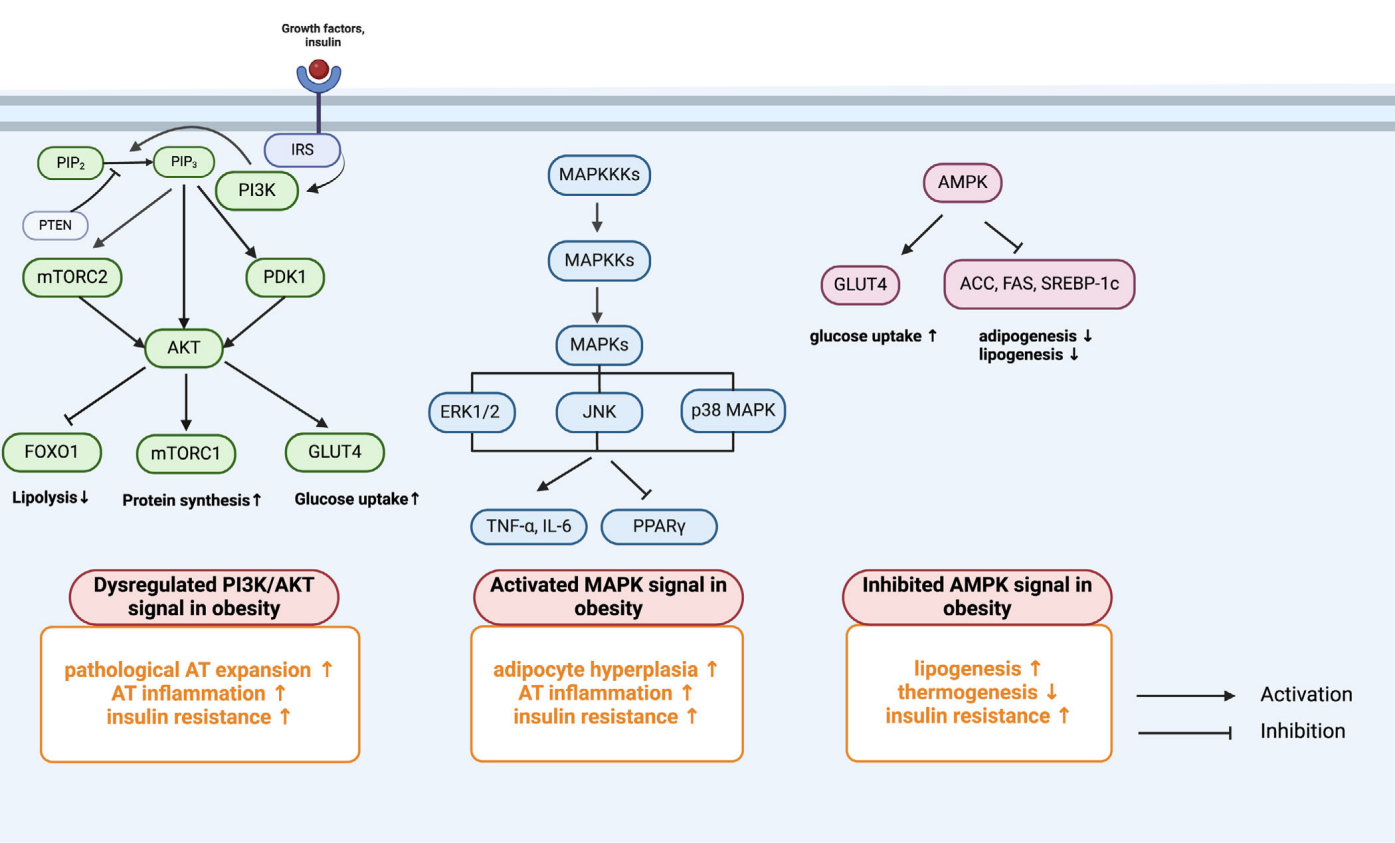
Potential signaling pathways and their roles in WAT during obesity. Under normal physiological conditions, the PI3K/AKT pathway activates GLUT4 and mTORC1 to increase glucose uptake and protein synthesis but inhibits lipolysis through FOXO1. The PI3K/AKT pathway is dysregulated in obesity, causing unhealthy AT expansion and inflammation, leading to insulin resistance. The MAPK signaling pathway is abnormally activated in obesity. Activation of MAPK signaling proteins, such as ERK1/2, JNK, and p38 MAPK, results in downstream regulation of TNFα, IL‐6, and PPARγ, leading to adipocyte hyperplasia, AT inflammation, and insulin resistance. The AMPK signaling pathway is inhibited in obesity. Thus, activation of AMPK may increase glucose uptake and decrease adipogenesis and lipogenesis, thereby improving insulin sensitivity and ameliorating obesity. ACC, acetyl‐CoA carboxylase; FAS, fatty acid synthesis; SREBP‐1c, sterol regulatory element‐binding protein 1c.

**FIGURE 5:**
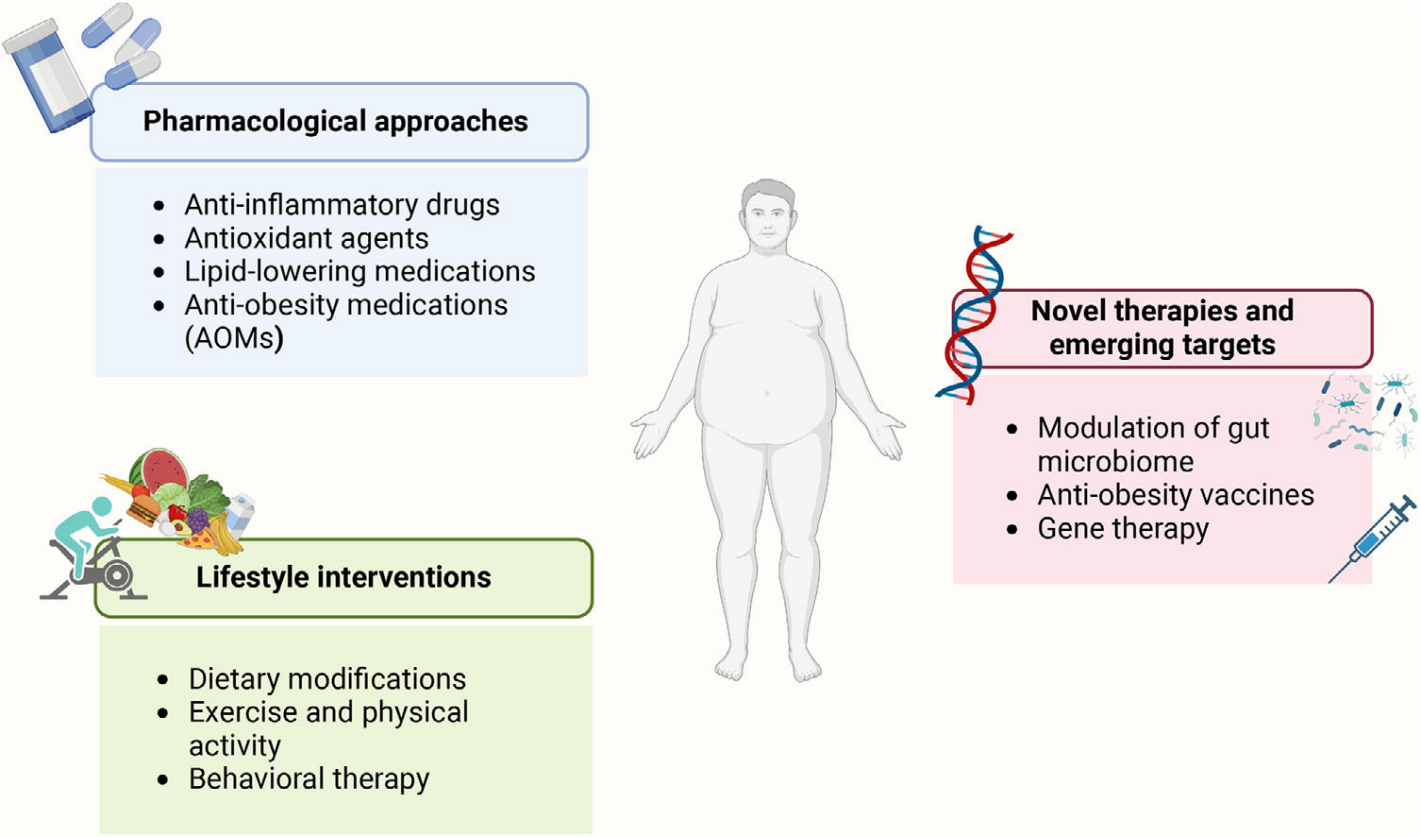
Potential antiobesity strategies. The figure outlines the potential antiobesity strategies discussed in the review, including pharmacological approaches, lifestyle interventions, and novel therapies and emerging targets.

The control of obesity has always been a great problem challenging our health system. Although lifestyle management, including caloric restriction and physical activity (PA), is first recommended for treating obesity, it has been proven to be insufficient and to provide moderate efficacy.^220^ Currently, most guidelines recommend that treatment strategies for obesity be promoted with the addition of pharmacotherapy and/or bariatric surgery.^221^, ^222^ Since AT is primarily afflicted by obesity, targeting AT may provide new insights into developing novel treatments for obesity. Here, we outline potential therapeutic signaling pathways and existing therapeutic strategies for ameliorating AT dysfunction in individuals with obesity (Figures 4 and 5).

### 4.1 Potential therapeutic signaling pathways

#### 4.1.1 PI3K/AKT signaling pathway

The phosphatidylinositol 3‐kinase (PI3K)/protein kinase B (AKT) pathway plays a crucial role in the growth, proliferation and metabolic homeostasis of insulin‐sensitive tissues, including AT, liver, and pancreas, and dysregulation of this signaling pathway leads to the development of obesity and T2D.^223^, ^224^, ^225^ After being activated by upstream signals, such as growth factors and insulin, PI3K transforms phosphatidylinositol 4,5‐bisphosphate to generate phosphatidylinositol 3,4,5‐trisphosphate, subsequently activating downstream effectors, including phosphoinositide‐dependent protein kinase 1, AKT, and the mammalian target of rapamycin complex (mTORC), to modulate several transcription factors that can enhance glucose uptake and inhibit lipolysis, such as forkhead box protein O1 (FOXO1), GLUT4, peroxisome proliferator‐activated receptors (PPARs), PPARγ coactivator‐1 alpha (PGC1α), and sterol regulatory element‐binding proteins (SREBPs).^226^, ^227^, ^228^, ^229^ Under conditions of excessive energy intake, the PI3K/AKT pathway is suppressed, causing increased lipolysis and decreased glucose uptake in AT, further aggravating circulating FFAs and leading to ectopic lipid accumulation and glucose metabolism imbalance.^224^ Moreover, the PI3K signaling pathway is related to AT inflammation by recruiting inflammatory cells. It has been reported that macrophage‐intrinsic PI3K signaling promotes metabolic health by driving ATM programs that are associated with the MARCO‐dependent uptake of lipids.^230^ Moreover, PI3Kγ knockout mice exhibit improved systemic insulin sensitivity and reduced HFD‐induced inflammation with decreased M1 macrophage infiltration.^231^ Furthermore, inhibition of PI3Kγ by using the PI3Kγ inhibitor AS‐605240 ameliorates IR and the abundance of ATMs in obese diabetic mouse models.^231^ Collectively, these findings demonstrated that the PI3K/AKT signaling pathway is highly important for preventing obesity‐induced inflammation and IR and could be a potential therapeutic target for treating obesity and obesity‐related metabolic disorders.

#### 4.1.2 MAPK signaling pathway

The MAPK signaling pathway, which includes ERK 1/2, JNK, and p38 MAPK, is a central mediator of the development of obesity and inflammation‐induced IR.^232^, ^233^ ERK1/2 activation has detrimental effects, such as enhancing lipolysis and inducing inflammation, IR and obesity.^234^ Mice lacking Erk1 (ERK1−/−) exhibit decreased adiposity and are protected from the development of IR and obesity during HFD feeding.^235^ Similarly, the activity of JNK is abnormally elevated in AT during obesity and T2D in different models.^236^, ^237^ In AT, JNK1 knockout mice are insulin sensitive, and the expression of the inflammatory cytokine IL6 is prevented by HFD feeding, which is mediated by downregulation of the expression of the IL‐6 target gene suppressor of cytokine signaling 3 (SOCS3).^238^ Another study reported that IR was prevented in mice with macrophage‐specific ablation of JNK, which was associated with reduced tissue infiltration by proinflammatory macrophages.^239^ Together, the activation of MAPKs is related to inflammatory cell infiltration, adipocyte hyperplasia, and IR.^240^ Integrated multiomic analysis also revealed that MAPK signaling cascades are activated in AT and are involved in inflammation‐associated energy metabolism upon HFD treatment.^241^ Indeed, several inhibitors targeting the MAPK pathway have provided therapeutic benefits, especially in inflammatory diseases and cancers.^242^, ^243^ Therefore, targeting these kinases may be a promising approach for treating metabolic disorders such as obesity and T2D.

#### 4.1.3 AMPK signaling pathway

Adenosine monophosphate (AMP)‐activated protein kinase (AMPK) plays a key role in the regulation of cellular and systemic energy balance^244^ and is considered a therapeutic target for the treatment of obesity and other metabolic diseases.^245^ Activation of AMPK by phosphorylation inhibits adipogenesis and lipogenesis through inactivation of PPARγ, C/EBPα, acetyl‐CoA carboxylase (ACC), fatty acid synthesis products, and SREBP‐1c in AT.^246^, ^247^ Activation of AMPK increases thermogenesis and energy expenditure in the WAT of mice fed a HFD.^248^ Individuals who are insulin resistant exhibit uniformly decreased AMPK activity in AT.^249^ Thus, stimulating AMPK in AT through various means such as norepinephrine or β3‐adrenergic agonists,^250^ AMPKβ1 activators (A‐769662)^251^ or genetic mutations (D316A mutation in AMPK‐γ1)^252^ enhances mitochondrial fatty acid oxidation and energy expenditure and has shown protective effects against diet‐induced obesity in mice fed a HFD. Furthermore, metformin, the first‐line drug for treating obesity‐related T2D, activates AMPK and suppresses TGF‐β1/Smad3 signaling, suppressing abnormal ECM remodeling in WAT and ameliorating IR in individuals with obesity.^253^

Taken together, the abovementioned signaling pathways play complicated roles in regulating the function of AT in individuals with obesity. Although considerable progress has been made in revealing the pathogenesis of obesity, it is still challenging for us to develop personalized treatment strategies by targeting specific signals/pathways in individuals with obesity because of the complexity of signal transduction pathways. Therefore, a better understanding of the molecular mechanisms of AT dysfunction will open up new avenues for understanding obesity and providing potential therapeutic approaches for treating this disease.

### 4.2 Pharmacological approaches

#### 4.2.1 Anti‐inflammatory drugs

Anti‐inflammatory agents have been identified as potential alternative treatments for obesity. Chronic low‐grade inflammation is a hallmark of dysfunctional AT in individuals with obesity and contributes to IR and other metabolic abnormalities. A systematic review of experimental studies revealed several anti‐inflammatory agents that act in metabolic pathways to reduce the expression of inflammatory cytokines, decrease macrophage infiltration in AT, and promote the polarization of M1 macrophages to M2 macrophages.^254^ Hsieh et al.^255^ showed that the administration of a selective COX‐2 inhibitor, such as celecoxib or mesulid, led to a substantial reversal of adipocyte hypertrophy, macrophage infiltration, and alterations in the genetic expression of TNF‐α, PPAR‐γ, and CCAAT‐enhancer‐binding proteins (C/EBP‐α) in the epididymal AT of rats. Ma et al.^256^ demonstrated that the drug dextran (D‐70) effectively targets adipose macrophages in obese mice, leading to a reduction in the production of MCP‐1,TNF‐α, and IL‐6 via the NF‐κB pathway, ultimately resulting in decreased inflammation in adipocytes. In a study by Furuya et al.,^257^ atorvastatin effectively reduced the phosphorylation of both IKK‐β and IKK‐α, leading to decreased expression of NF‐κB target genes such as TNF‐α and IL‐6 in obese mice. Additionally, there was an increase in both the gene and protein expression of GLUT4, a GLUT involved in insulin sensitivity. However, the absence of effective agents underscores the necessity to comprehensively evaluate the underlying mechanisms at play and pinpoint suitable therapeutic targets.

#### 4.2.2 Antioxidant agents

In addition to pharmaceuticals, several bioactive compounds derived from plants and synthetic sources are commonly used for treating obesity. These compounds possess anti‐inflammatory properties and play a significant role in the treatment of obesity through their antioxidant and anti‐inflammatory effects, which help reduce ROS levels and mitigate inflammatory responses.^258^, ^259^ Alsaggar et al.^260^ investigated the anti‐inflammatory and antioxidant properties of Silibinin. Their study revealed that Silibinin mitigates inflammation in AT and effectively counteracts obesity and its associated complications in a mouse model of diet‐induced obesity.^260^ Gao et al.^261^ documented that rutin, a potent natural antioxidant, inhibits the infiltration and clustering of macrophages around necrotic adipocytes, which leads to a reduction in adipocyte hypertrophy and mitigates the formation of CLSs, consequently alleviating chronic inflammation.

#### 4.2.3 Lipid‐lowering medications

Lipid‐lowering medications are a class of drugs that are used to reduce the blood lipid level. These medications are commonly used to treat hyperlipidemia, which is a condition characterized by high levels of cholesterol and/or TGs in the blood.^262^ A systematic review of experimental studies revealed that statins, a type of lipid‐lowering medication, can reduce the expression of inflammatory cytokines, decrease macrophage infiltration in AT, and promote the polarization of M1 macrophages to M2 macrophages.^263^ Other lipid‐lowering agents, such as bempedoic acid, inclisiran, icosapent ethyl, pemafibrate, and RNA‐based therapies, have also shown promising results in reducing the cardiovascular burden in patients at highest risk.^263^ However, the area of lipid‐modulating agents is still ripe, and major novelties need to be addressed in the next few years.

#### 4.2.4 Antiobesity medications

With advances in technology and pharmaceutics, various antiobesity medications (AOMs) have been developed for long‐term weight management; these agents target different factors and signaling pathways.^264^ However, AOMs predominantly function via central nervous system (CNS) mechanisms to increase satiety and decrease food intake, which are safety concerns due to their adverse cardiovascular effects, increased suicidal risk, and so on.^265^ Therefore, it is important to develop safe and effective prevention strategies and remedies for obesity.

TZDs are among the primary antidiabetic drugs used to treat obese T2D patients. The TZD drug family members, including rosiglitazone and pioglitazone, are potent PPARγ agonists that can promote adipogenesis, improve insulin sensitivity, and enhance glucose utilization in WAT.^266^, ^267^ Recently, several studies have demonstrated that novel TZD derivatives not only activate PPARγ but also inhibit PTP1B in diabetic mouse models.^268^, ^269^, ^270^ PTP1B, expressed in multiple cell types including the liver, muscle, and AT, serves as a critical negative regulator of insulin and leptin signaling pathways, including the PI3K/Akt and JAK2/STAT3 cascades.^271^ Hence, PTP1B has been suggested as a potential therapeutic target for the management of diabetes, obesity, and other associated metabolic disorders.^272^ Among numerous different PTP1B inhibitors, trodusquemine (MSI‐1436) has been found to cause fat‐specific weight loss and improve insulin and leptin levels in DIO mouse models.^273^ Moreover, a phase I clinical trial is currently being carried out in diabetic and/or obese patients (https://clinicaltrials.gov: NCT00509132, NCT00806338, and NCT00606112).

GLP‐1 receptor agonists are primarily used for the treatment of T2D but have also demonstrated weight loss effects. They increase insulin secretion, delay gastric emptying, and promote satiety, leading to reduced food intake and potential WAT remodeling.^274^ In addition to the complicated mechanism of GLP‐1/GLP‐R in the CNS, GLP‐1 also plays an important role in improving insulin sensitivity in AT, partially through AMPK‐related pathways.^275^ Numerous in vitro investigations have unveiled that GLP‐1 signaling acts as a regulator of adipogenesis. Activation of GLP‐1R induces the upregulation of differentiation marker genes such as PPARγ and FABP4, thereby promoting lipid accumulation during preadipocyte differentiation.^276^, ^277^ Liraglutide (Saxenda) and semaglutide (Wegovy) are GLP‐1R agonists approved by the United States Food and Drug Administration for obesity treatment in 2014 and 2021, promoting the belief that breakthrough, drug‐based management of obesity may be possible.^264^, ^278^, ^279^ Ongoing research needs to continue to explore new drug targets and therapies aimed at modulating WAT metabolism to combat obesity and related metabolic disorders.

### 4.3 Lifestyle interventions

Lifestyle interventions are a key approach to managing obesity. They typically involve changes in diet, PA, and behavior.^222^ Dietary modifications involve making changes to the diet to promote weight loss and improve overall health. This may include reducing caloric intake, controlling portions of food, choosing nutrient‐dense foods, increasing fiber intake, limiting processed foods and sugary beverages, and adopting balanced eating patterns such as Mediterranean or DASH (dietary approaches to stop hypertension) diets.^280^ In recent years, intermittent fasting has surged in popularity and has demonstrated generally favorable effects on health. Nevertheless, the advantages and hurdles, particularly regarding acceptance and compliance, of long‐term fasting still necessitate additional research for comprehensive understanding.^281^

Regular PA is also essential for weight management and improving metabolic health. Participating in PA and exercise training (ET) has been associated with reduced cardiovascular risk, improved cardiometabolic risk factors, and enhanced weight loss by creating a caloric deficit. The most effective strategy for promoting weight loss involves a combination of caloric restriction and maintaining appropriate levels of aerobic PA/ET.^282^

Behavioral therapy focuses on changing habits, attitudes, and behaviors related to eating and PA. Although behavioral therapy has a modest effect on weight loss [−1.7 kg (95% CI −2.52 to −0.86)] (99), instituting behavioral therapy with lifestyle modification amplifies weight loss [−4.9 kg (95% CI −7.3 to −2.4)].^283^, ^284^

Overall, the treatment of obesity requires multidimensional therapeutic interventions under the guidance of multiple clinical professionals. There is no one‐size‐fits‐all approach. Clinicians may also need to combine medication therapy, lifestyle modifications, and behavioral therapy with bariatric surgery to achieve the best treatment outcomes.

### 4.4 Novel therapies and emerging targets

Recent advances in understanding the pathophysiology of obesity have uncovered several promising drug targets and innovative therapeutic approaches. These discoveries offer potential solutions to combat the worldwide obesity epidemic and its associated health complications. In addition to pharmacotherapeutics, alternative strategies for combating obesity, including modulation of the gut microbiome, antiobesity vaccines and gene therapy, are being explored.

Obesity has been linked to changes in the composition of the gut microbiota, such as an elevated ratio of Firmicutes to Bacteroidetes,^285^ decreased microbial diversity, and reduced richness of microbial genes.^286^ The gut microbiome is emerging as a novel target for counteracting obesity. Therapeutic modulation of the gut microbiota could prevent and/or treat obesity and obesity‐associated metabolic disorders.^287^ Selective modulation of the human gut microbiome is an innovative approach for treating obesity. This can be accomplished through dietary supplementation with prebiotics and probiotics, which can influence bacterial growth. Another method is fecal microbiota transplantation.

Oral immunization via the use of antigens derived from AT has been employed to induce immune tolerance to self‐antigens, which is considered safe but lacks specificity. Although the impact on body weight has been minimal, human studies have shown that this strategy results in reduced waist circumference and improved lipid metabolism.^288^ Studies have demonstrated that antisomatostatin vaccination can lead to a 10% reduction in body weight gain in diet‐induced obese mice but has no impact on energy intake.^289^

The occurrence of obesity arises from the complex interplay between genetic, epigenetic, developmental, and environmental factors. Gene therapy for obesity aims to restore and maintain energy homeostasis by effectively delivering and expressing therapeutic genes in specific cells.^290^ Gene therapy predominantly employs viral vector delivery systems, nonviral gene carriers like proteins and lipids, and genome‐editing technologies such as zinc finger nucleases, clustered regularly interspaced short palindromic repeats (CRISPR) systems, and transcription activator‐like effector nucleases.^291^ These methods play a significant role in gene therapy approaches. An interesting study in diet‐induced obese mice targeted the delivery of the CRISPR interference system against Fabp4 to white adipocytes, which reduced body weight by an astonishing 20%1 and ameliorated inflammation, hepatic steatosis, and IR.^292^

## 5 CONCLUSIONS

Dysfunction of AT is highly important in the development of obesity and obesity‐related metabolic diseases. As WAT is a highly heterogeneous organ, a variety of cells are involved in the progression of obesity and metabolic dysfunction. Initially, adipocyte hypertrophy occurs, where existing fat cells increase in size due to excess lipid accumulation, and hypertrophic adipocytes exacerbate inflammation by secreting cytokines to recruit immune cells, dysregulating the adipogenic potential of PreAs, accumulating proinflammatory immune cells and reducing the number of anti‐inflammatory immune cells. The complicated interaction between different cells further promotes inflammation and dysfunction in AT. Additionally, there is a shift in WAT macrophage polarization toward the proinflammatory M1 phenotype. As obesity progresses, adipogenesis and adipocyte differentiation capacity decrease, while fibrosis and collagen deposition increase within the WAT microenvironment. These cellular changes collectively contribute to the dysregulation of WAT function and IR observed in individuals with obesity.

Pathological expansion of AT leads to ectopic lipid accumulation and hypoxia; promotes lipotoxicity, AT fibrosis, and proinflammatory immune cell infiltration; and ultimately results in an inflammatory phenotype and associated immunometabolic dysfunction. Dysfunction of WAT during long periods of overnutrition eventually leads to systemic inflammation and IR. Local inflammation in WAT triggers the secretion of inflammatory adipokines, cytokines, and chemokines and subsequently negatively affects remote organs such as muscle, liver, and heart, leading to IR and causing complications associated with obesity. Obesity‐induced inflammation activates various signaling pathways, impairs insulin signaling, and promotes the release of proinflammatory mediators such as TNF‐α, IL‐1β, and IL‐6, all of which contribute to IR. Additionally, obesity‐related IR involves pancreatic β‐cell dysfunction due to chronic exposure to high FFAs and hyperinsulinemia. Understanding these complex dynamics is crucial for developing targeted interventions against the metabolic consequences of obesity.

Obesity is a challenging problem, and current lifestyle management strategies are often insufficient. The treatment of obesity often includes pharmacotherapy and/or bariatric surgery in addition to lifestyle changes. AT dysfunction plays a key role in obesity, and targeting AT may lead to new treatment approaches. Several signaling pathways, including the PI3K/AKT, MAPK, and AMPK pathways, have been identified as potential therapeutic targets. These pathways regulate AT growth, inflammation, and insulin sensitivity. Additionally, various drugs have been developed to target AT metabolism in individuals with obesity. GLP‐1 receptor agonists promote satiety and weight loss and have shown great success in people suffering from obesity, ushering in a “new dawn” for obesity treatment. However, further research is needed to explore new drug targets and therapies for combating obesity and related metabolic disorders.

Therefore, a deep understanding of the pathogenesis mechanisms at different levels that lead to AT dysfunction is strongly needed. Notably, advances in science and technology have allowed us to evaluate obesity at the tissue, cellular, and molecular levels. With further advancements in comprehending the pathophysiological foundation and individual variations of obesity, personalized, multimodal approaches to obesity treatment are anticipated to emerge. These approaches aim to facilitate safe, effective, and long‐lasting weight loss, subsequently leading to a reduced prevalence of obesity and obesity‐related comorbidities.

## AUTHOR CONTRIBUTIONS

**Zi‐Han Yang; Fang‐Zhou Chen; Yi‐Xiang Zhang**; and **Min‐Yi Ou**: Wrote the manuscript and generated the figures. **Poh‐Ching Tan**: Supervised the manuscript and modified the figures. **Xue‐Wen Xu**: Provided a critical review and helped edit the manuscript. **Qing‐Feng Li** and **Shuang‐Bai Zhou**: Conceived the idea and supervised the manuscript. All the authors read and approved the final manuscript.

## CONFLICT OF INTEREST STATEMENT

The authors declare that they have no conflict of interest.

## ETHICS STATEMENT

Not applicable.

## Data Availability

Not applicable.
